# Patient Characteristics and Product Use Behaviors Among Persons with E-cigarette, or Vaping, Product Use–Associated Lung Injury — Indiana, June – October 2019

**DOI:** 10.15585/mmwr.mm6849a3

**Published:** 2019-12-13

**Authors:** Kathryn L. Gaub, Sara Hallyburton, Claudine Samanic, Deanna Paddack, Charles R. Clark, Shawn Pence, Jennifer A. Brown, Eric Hawkins

**Affiliations:** ^1^Epidemic Intelligence Service, CDC; ^2^Indiana State Department of Health; ^3^Division of Population Health, National Center for Chronic Disease Prevention and Health Promotion, CDC.

As of December 4, 2019, a total of 2,291 cases of hospitalized e-cigarette, or vaping, product use–associated lung injury (EVALI) have been reported from 50 states, the District of Columbia, and two U.S. territories (Puerto Rico and the U.S. Virgin Islands) (*1*). State health departments, including the Indiana State Department of Health (ISDH), are working with their local health departments and with CDC, the Food and Drug Administration, and other clinical and public health partners in investigating this outbreak of EVALI. On August 7, 2019, ISDH issued an advisory regarding patients hospitalized in Wisconsin with severe acute lung injury who reported the use of e-cigarette, or vaping, products (*2*); health care providers were requested to notify ISDH of similar cases. On August 8, 2019, ISDH received reports of five similar cases among Indiana residents. Suspected cases EVALI reported to ISDH were investigated further only among patients who required hospitalization. Established case definitions were used to classify cases.* Medical record abstractions and patient interviews were completed using nationally standardized forms to ascertain patient characteristics, medical care received, and product-use behaviors.

A total of 127 suspected EVALI cases were reported to ISDH during August 8–October 28, 2019; among these, 97 (76%) patients met the confirmed (41; 42%) or probable (56; 58%) case definitions and were hospitalized, including three (3%) who died. Because of staffing constraints, medical record abstractions could only be completed for 54 (56%) patients; among these, the median age was 26 years (range = 16–68 years), and 38 (70%) were male (Table 1). Among the patients for whom information on medical care was available, 13 of 51 (25%) were admitted to an intensive care unit, 34 of 52 (65%) were treated with steroids, and seven of 50 (14%) required intubation and mechanical ventilation. Among the 54 patients with available information documenting preexisting conditions, nine (14%) had asthma, 12 (22%) had depression, and 14 (26%) had anxiety.

**TABLE 1:** Characteristics of patients hospitalized with e-cigarette, or vaping, product use–associated lung injury ascertained from medical record abstraction (N = 54) — Indiana, June–October 2019

Patient characteristic (no. with available information)	No. (%*)
**Sex (54)**	
Male	38 (70)
Female	16 (30)
**Age group (yrs) (54)**	
13–17	7 (13)
18–29	27 (50)
30–39	12 (22)
40–49	3 (6)
50–59	3 (6)
≥60	2 (4)
**Symptoms on admission (54)**	
Shortness of breath	48 (89)
Cough	44 (81)
Chest pain	17 (31)
Nausea	27 (50)
Vomiting	27 (50)
Diarrhea	15 (28)
Abdominal pain	12 (22)
Weight loss	8 (15)
Sweating	11 (20)
**Preexisting conditions (54)**	
Asthma	9 (17)
COPD	2 (4)
Depression	12 (22)
Anxiety	14 (26)
**Medical care received**	
Antibiotics (51)	44 (86)
Steroids (52)	34 (65)
Bronchoscopy (44)	13 (30)
Lung biopsy (45)	7 (16)
ICU admission (51)	13 (25)
Intubation and mechanical ventilation (50)	7 (14)

**Abbreviations:** COPD = chronic obstructive pulmonary disease; ICU = intensive care unit.

* Percentages rounded and therefore might not sum to 100% in each category.

Interviews were successfully completed for 29 (30%) of 97 patients (Table 2); 33 (34%) patients were lost to follow-up or refused to be interviewed. Among the 29 patients interviewed, seven (24%) reported using only tetrahydrocannabinol (THC)-containing products, seven (24%) reported using only nicotine-containing products, 13 (45%) reported using both, and two (7%) reported using flavored products containing neither THC nor nicotine. A total of 20 (69%) reported using any THC-containing product; among those with available information on product use, eight of 15 (53%) reported using the product daily, 14 of 19 (74%) reported using products labeled “Dank Vapes,” and 10 of 20 (50%) obtained the products from a friend or other acquaintance.

**TABLE 2:** Product use preferences and behaviors of patients hospitalized with e-cigarette, or vaping product use–associated lung injury identified through patient interview (N = 29) — Indiana, June–October 2019

**Product preference/behavior (no. with available information**	No. (%*)
**Type of product (29)**	
THC-containing only	7 (24)
Nicotine-containing only	7 (24)
Both THC- and nicotine-containing	13 (45)
Neither THC- nor nicotine-containing	2 (7)
**Nicotine-containing brands used (19)**	
Juul	8 (42)
Njoy	2 (11)
**Nicotine-containing product use frequency (19)**	
Daily	16 (84)
2–3 times per week	2 (11)
Monthly or less	1 (5)
**THC-containing brands used (19)**	
Dank Vapes	14 (74)
Chronic Carts	2 (11)
Exotic Carts	2 (11)
**THC-containing product use frequency (15)**	
Daily	8 (53)
2–3 times per week	3 (20)
Monthly or less	4 (27)
**Source of THC-containing products (20)**	
Friend or acquaintance	10 (50)
Online	1 (5)

**Abbreviation:** THC = tetrahydrocannabinol.

* Percentages rounded and therefore might not sum to 100% in each category.

The percentage of Indiana EVALI patients who reported using THC-containing products (69%) was lower than that reported by patients in Utah (92%), Illinois and Wisconsin (80%), and nationally (80%) (*1*–*3*). Reported concurrent use of THC-containing and nicotine-containing products in Indiana (45%) was also slightly lower than that reported nationally (52%) (*1*). Further, nearly one third (31%) of Indiana patients reported using products that did not contain THC. These findings need to be investigated to determine whether Indiana patients might have underreported THC use or whether use of multiple product types might cause EVALI. Results of this investigation of Indiana EVALI patients were consistent with findings from Illinois that frequently using THC-containing products and obtaining these products through personal contacts has been associated with EVALI (*4*). In addition, the high proportion of reported use of Dank Vape products might be important, because these products are largely counterfeit (*4*).

The findings in this report are subject to at least four limitations. First, medical record abstractions were completed for one half of EVALI patients in Indiana, and only one third were interviewed, which might result in selection bias and inaccurate estimates of patient characteristics and product use patterns. Second, preexisting conditions and details of medical care were not consistently documented in medical records. Therefore, percentages reported here could be underestimates. Third, because THC-containing products are illegal in Indiana, use of these products might be underreported for fear of legal repercussions or perceived stigma. Finally, only hospitalized cases in Indiana were investigated, potentially leading to bias related to the severity of cases.

The specific cause, or causes, of EVALI has not yet been determined. Vitamin E acetate, frequently used as a thickening agent in THC-containing products, has been detected in bronchoalveolar lavage samples from 29 EVALI patients in 10 states and five EVALI patients in Minnesota (*5*,*6*). However, it is possible that more than one compound or ingredient could be the cause, and evidence is not yet sufficient to rule out contributions of other toxicants to EVALI. CDC recommends not using e-cigarette, or vaping, products containing THC, especially those obtained from informal sources such as friends, family, or in-person or online dealers (*1*). Because the specific cause or causes of lung injury are not yet known, persons should consider refraining from use of all e-cigarette, or vaping, products (*1*). Youths, young adults, or women who are pregnant should never use e-cigarette, or vaping, products (*1*).

**Summary**

**What is already known about this topic?**

As of December 4, 2019, 2,291 U.S. persons have been hospitalized with cases of e-cigarette, or vaping, product use–associated lung injury (EVALI). Among those with available data, 80% reported using products containing tetrahydrocannabinol (THC).

**What is added by this report?**

During August 8–October 28, 2019, 97 patients in Indiana were hospitalized with confirmed or probable cases of EVALI. Interviews were completed for 29 patients; among these, 69% reported using THC-containing products.

**What are the implications for public health practice?**

The percentage of Indiana EVALI patients reporting THC use was low compared with that reported for all U.S. EVALI patients. This might be due to underreporting of THC use but could also suggest that EVALI is not exclusively associated with THC use.

## Footnotes

* https://www.cdc.gov/tobacco/basic_information/e-cigarettes/assets/2019-Lung-Injury-Surveillance-Case-Definition-508.pdf.
